# Slow Dissolution Kinetics of Model Peptide Fibrils

**DOI:** 10.3390/ijms21207671

**Published:** 2020-10-16

**Authors:** Mona Koder Hamid, Axel Rüter, Stefan Kuczera, Ulf Olsson

**Affiliations:** Division of Physical Chemistry, Lund University, P.O. Box 124, 22210 Lund, Sweden; mona.koder_hamid@teokem.lu.se (M.K.H.); stefan.kuczera@gu.se (S.K.); ulf.olsson@fkem1.lu.se (U.O.)

**Keywords:** dissolution kinetics, self-assembly, peptides

## Abstract

Understanding the kinetics of peptide self-assembly is important because of the involvement of peptide amyloid fibrils in several neurodegenerative diseases. In this paper, we have studied the dissolution kinetics of self-assembled model peptide fibrils after a dilution quench. Due to the low concentrations involved, the experimental method of choice was isothermal titration calorimetry (ITC). We show that the dissolution is a strikingly slow and reaction-limited process, that can be timescale separated from other rapid processes associated with dilution in the ITC experiment. We argue that the rate-limiting step of dissolution involves the breaking up of inter-peptide β–sheet hydrogen bonds, replacing them with peptide–water hydrogen bonds. Complementary pH experiments revealed that the self-assembly involves partial deprotonation of the peptide molecules.

## 1. Introduction

Peptide self–assembly has been studied extensively in the last decades. One reason is that peptide aggregation into long fibrillar structures, often referred to as amyloids, is a hallmark of a number of neurodegenerative diseases [1,2,3], including Alzheimer’s and Parkinson’s. However, there is also a considerable interest within the materials science community [4,5,6] and within the area of peptide-based drugs [7,8]. Most of the studies deal with the characterization of the structures formed at steady state [4,9,10], but there are also a number of studies that address the kinetics of self–assembly [11,12,13,14].

In order to understand the consequences of amyloid formation in vivo, we also need to address the molecular pathways and kinetics of fibril assembly and disassembly. Recent developments of experimental protocols [11] have allowed for the collection of accurate and reproducible kinetic data of fibril formation. Based on these data, a kinetic model for the fibril formation process could be developed [15], taking into account both primary and secondary nucleation processes. The nucleation process may also involve disordered complexes [16] and well-defined oligomer states [15,17]. It has also recently been suggested that some molecular pathways may be intrinsically catalytic in their nature, such that they can display saturation effects [18]. The reverse process, disassembly or aggregate dissolution, has been less studied. Dissolution kinetics can be of interest for certain applications, e.g., drug administration, and it is interesting to understand assembly and disassembly kinetics from the point of view of microscopic reversibility.

**Figure 1:**
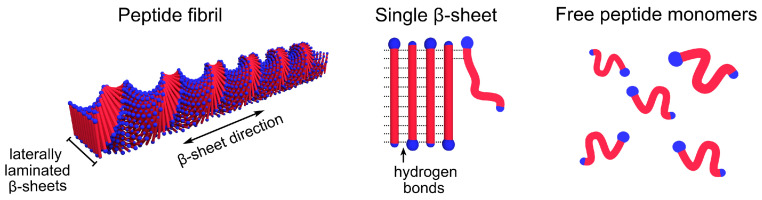
A schematic visualization of an A${}_{8}$K peptide fibril and its constituents.

Amyloid-forming peptides like Amyloid–β (Aβ) or α-Synuclein essentially fold in two dimensions when aggregating into fibrils. More or less complex conformational changes may, therefore, significantly slow down the steps in the pathway of aggregation. Thus, it can be useful to simplify the system by studying short model peptides, where the self-assembled fibrils can have a simpler molecular packing. One such model system, for which the self-assembly structure has been extensively characterized, is the alanine-rich peptide A${}_{8}$K, where A denotes alanine and K is lysine [19,20]. Above a well-defined peptide concentration in water, which can be seen as the monomer solubility, $c_{s}$, A${}_{8}$K self–assembles, driven by the hydrophobic interaction, into twisted ribbon aggregates. These aggregates are of the order of 100–200 nm long and consist of circa 15 laminated β–sheets. A schematic representation of the aggregates is shown in Figure 1. They have an approximately rectangular cross-section of 4×8 $nm^{2}$, where the shorter dimension corresponds to the peptide length. The longer dimension, 8 nm, is the result of 15 laterally laminated β-sheets, separated by roughly 0.5 nm [21].

In this paper, we address the dissolution kinetics of A${}_{8}$K ribbons, as the aggregates are diluted below $c_{s}$. Due to the low concentrations involved, detecting the dissolution by, e.g., scattering techniques, is difficult, and we here have instead explored the use of isothermal calorimetry, ITC, as a probe to monitor dissolution through the heat released in the process. ITC is a useful technique to investigate the thermodynamics of self-assembly, and has been used extensively to characterize, e.g., surfactant micelle formation, see, e.g., reference [22,23] and further references therein. However, there are only a few reports where this technique was applied to study the aggregation of peptides [24,25,26].

## 2. Results and Discussion

The monomer solubility for the present A${}_{8}$K batch was determined to $c_{s}=5$ mM using static light scattering (SLS), following a previous protocol [19]. The scattered intensity was measured as a function of the peptide concentration, *c*, when gradually diluting a stock solution of concentration $c>c_{s}$.

**Figure 2:**
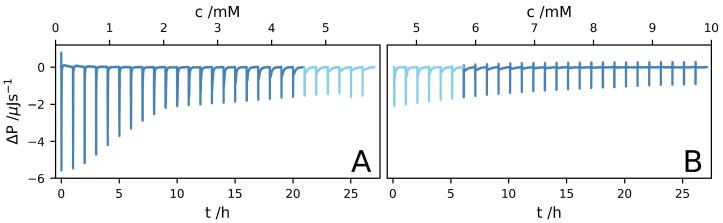
Isothermal titration caliometry (ITC) data, plotted as the differential power, ΔP(t), versus time, t, and A${}_{8}$K concentration. Two datasets, (**A**,**B**), are shown. In (**A**), the sample cell contained initially pure D${}_{2}$O, while in (**B**), the sample cell contained initially a 4.4 mM A${}_{8}$K solution. The data have been corrected for minor baseline drift.

Two ITC experiments, A and B, were performed. In experiment A, the sample cell initially contained pure D${}_{2}$O, while in experiment B, the sample cell initially contained a 4.4 mM A${}_{8}$K solution. The data from the two experiments are presented in Figure 2A,B, respectively, as the differential power ΔP versus time and A${}_{8}$K concentration. Each injection corresponds to a concentration increase of circa 0.21 mM. After the 27th injection of experiment A, the concentration in the cell reached 5.6 mM. In experiment B, the concentration varied from 4.6 mM after the first injection to 9.8 mM after the 27th injection. Thus, the peptide concentration reaches the solubility, 5 mM, around the 24th injection of experiment A, and roughly after three injections in experiment B.

**Figure 3:**
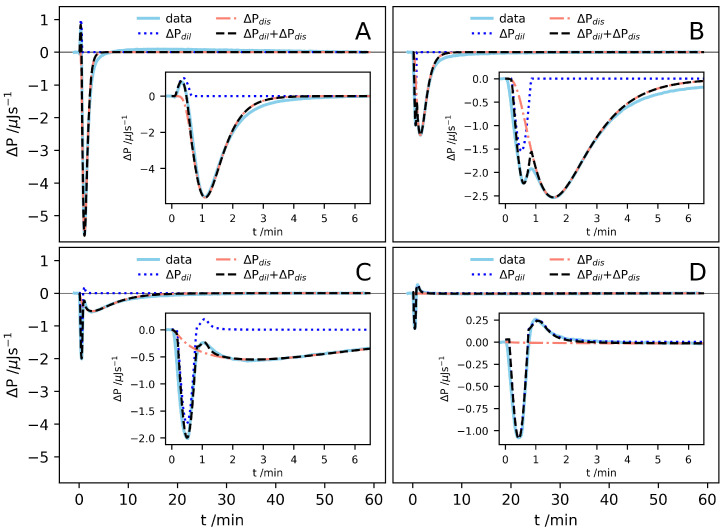
ΔP(t) traces from some selected injections. The different concentrations after injection are (**A**) c=0.28 mM, (**B**) c=1.9 mM, (**C**) c=4.9 mM and (**D**) c=8.5 mM. The ΔP(t) traces are shown together with the assumed functions for $\Delta P_{\mathrm{dis}}$, $\Delta P_{\mathrm{dil}}$ and the sum of the two. Inserts show a magnification of the same signal during the first minutes.

A closer inspection of the ΔP(t) signals after different injections reveal both fast and slow processes contributing to the observed enthalpy change. In Figure 3, some selected ΔP(t) traces are shown. In each injection, a 30 mM solution containing A${}_{8}$K aggregates is first diluted, resulting in an enthalpy change, $\Delta H_{\mathrm{dil}}$. In addition, if the concentration after injection is below $c_{s}$, the aggregates also dissolve, for which there is a corresponding enthalpy change $\Delta H_{\mathrm{dis}}$. Due to continuous stirring, the dilution process is expected to be fast, occurring essentially within the 30 s long injection. In some cases, two fast processes are resolved. The slow exothermic process observed at lower concentrations we thus associate with the dissolution of the peptide aggregates. With these two contributions. we can write $\Delta P\left(t\right)=\Delta P_{\mathrm{dil}}\left(t\right)+\Delta P_{\mathrm{dis}}\left(t\right)$, with $\Delta P_{\mathrm{dil}}\left(t\right)=dH_{\mathrm{dil}}/dt$ and $\Delta P_{\mathrm{dis}}\left(t\right)=dH_{\mathrm{dis}}/dt$, respectively.

The solution consists of peptides in aggregates, with the peptide concentration $c_{\mathrm{agg}}$, and free monomers of the concentration $c_{\mathrm{mon}}$, so that the total peptide concentration $c=c_{\mathrm{agg}}+c_{\mathrm{mon}}$. When the solution is diluted below $c_{s}$, the aggregates dissolve into monomers. During this process, we have $dc_{\mathrm{agg}}/dt=-dc_{\mathrm{mon}}/dt$. For a given peptide concentration, there is a given enthalpy change associated with every dissolved molecule and we expect $\Delta P_{\mathrm{dis}}∼dc_{\mathrm{mon}}/dt$. The dissolution process may be complex and involve more than a simple molecular detachment step. For example, nanotubes of the similar peptide A${}_{6}$K were found to dissolve in two steps. First, the laminated β–sheets in the aggregates were separated, followed by dissolution of the β–sheets into separate molecules [14]. Looking at the $\Delta P_{\mathrm{dis}}$ signals (Figure 3), however it seems that this can be described by a single process with a characteristic time that increases with increasing concentration. We see also that $\Delta P_{\mathrm{dis}}\left(t\right)$ shows a minimum at a time which is significantly longer than the injection time, 30 s. This implies that there is initial delay in the dissolution process.

To model the signal, we first note that the concentration, *c*, in the cell increases with the amount Δc(t) during the injection where, ideally,

(1) $$\Delta c\left(t\right)=\left\{\begin{array}{cc} \Delta c_{0}\frac{t}{t_{\mathrm{inj}}}, & \mathrm{for}\;t<t_{\mathrm{inj}}\\ \Delta c_{0}, & \mathrm{for}\;t\geq t_{\mathrm{inj}} \end{array}\right.$$

where $t_{\mathrm{inj}}=30$ s is the total injection time. Here, $\Delta c_{0}=c_{\mathrm{inj}}\left(V_{\mathrm{inj}}/V_{\mathrm{cell}}\right)$, with $c_{\mathrm{inj}}=30$ mM being the peptide concentration in the syringe, $V_{\mathrm{inj}}=10$ μL is the total injected volume and $V_{\mathrm{cell}}=1.46$ mL is the cell volume, which is constant throughout the experiment, so that $\Delta c_{0}=0.21$ mM. Below, however, we will approximate Δc(t) with an exponential function

(2) $$\Delta c\left(t\right)=\Delta c_{0}\left(1-e^{-k_{\mathrm{inj}}t}\right)$$

to avoid complications associated with a discontinuous first derivative. The rate constant is chosen as $k_{\mathrm{inj}}=1/15$ $s^{-1}$, giving a similar time dependence as in Equation (1). The aggregate concentration that is injected is expected to be $c_{\mathrm{inj}}-c_{s}$, assuming $c_{\mathrm{mon}}=c_{s}$ in the syringe. If the cell concentration is $c<c_{s}$, the injected aggregates will dissolve, producing monomers. The fact that there appears to be an initial lag time in the dissolution process indicates that the monomer production due to aggregate dissolution has a sigmoidal type time-dependence, and we assume the following test function

(3) $$c_{\mathrm{mon}}\left(t\right)=c_{\mathrm{mon}}\left(0\right)+\left(1-f_{\mathrm{agg}}\right)\Delta c\left(t\right)+f_{\mathrm{agg}}\Delta c\left(t\right){\left(1-e^{-kt}\right)}^{\alpha }$$

where the exponent α>1 gives an effective lag time. Here, $c_{\mathrm{mon}}\left(0\right)$ is the monomer concentration in the cell before injection and $\left(1-f_{\mathrm{agg}}\right)\Delta c\left(t\right)$ is the direct addition of monomers from the injection. $f_{\mathrm{agg}}=1-c_{s}/c_{\mathrm{inj}}$ is the fraction of the injected peptides that are in aggregates, and *k* is the dissolution rate constant. Assuming $\Delta P_{\mathrm{dis}}∼-dc_{\mathrm{agg}}/dt$, we then consider the derivative of the last term of Equation (3) after substitution for Δc(t) from Equation (2), and write

(4) $$\Delta P_{\mathrm{dis}}\left(t\right)=Af_{\mathrm{agg}}\Delta c_{0}\left[k_{\mathrm{inj}}e^{-k_{\mathrm{inj}}t}{\left(1-e^{-kt}\right)}^{\alpha }+\left(1-e^{-k_{\mathrm{inj}}t}\right)\alpha ke^{-kt}{\left(1-e^{-kt}\right)}^{\alpha -1}\right]$$

Here, *A* is a proportionality constant. The enthalpy is then given by

(5) $$\Delta H=\int_{0}^{\infty }dt\Delta P_{\mathrm{dis}}=Af_{\mathrm{agg}}\Delta c_{0}$$

The fast process(es), $\Delta P_{\mathrm{dil}}\left(t\right)$, can be modelled in a similar way. However, here we choose to focus only on the enthalpy and simply use a functional form for $\Delta P_{\mathrm{dil}}\left(t\right)$ that gives a reasonable description of the experimental data. It turned out that the shape of the dilution peaks, in most cases, could be well described by half a period of a sinusoidal function, $\Delta P_{\mathrm{dil}}=B\sin\left(\omega t-\delta \right)$, with *B*, ω and δ being variables regulating the signal amplitude, angular frequency and a delay. At higher concentrations, two separate $\Delta P_{\mathrm{dil}}$ signals are resolved: one endothermic and one exothermic. Here, the endothermic mode is better described by a Lorentzian, $\Delta P_{\mathrm{dil}}=C/\left[1+w{\left(t-\delta \right)}^{2}\right]$, where *C*, *w* and δ are constants regulating the amplitude, width and delay, respectively. To model the contributions arising from dilution, only the amplitude constants *B* and *C* were varied and constant values for ω and δ were used. All contributions to ΔP(t) were fitted by eye. In Figure 3, we also show the model calculations of $\Delta P\left(t\right)=\Delta P_{\mathrm{dil}}\left(t\right)+\Delta P_{\mathrm{dis}}\left(t\right)$, used to evaluate the corresponding enthalpies, together with the experimental data, for the four selected injections.

**Figure 4:**
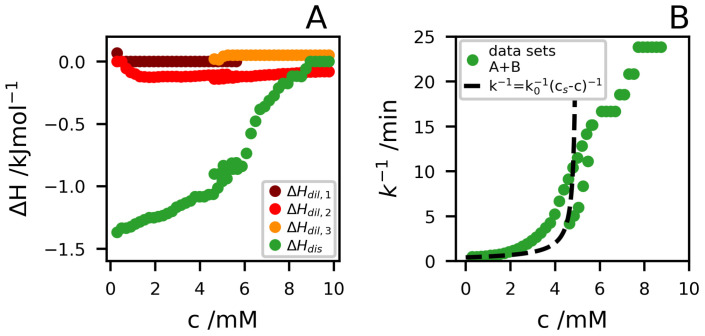
(**A**) ΔH as a function of *c* for all the detected processes. (**B**) $k^{-1}$ versus *c*. In both the figures data from datasets A and B are merged.

In Figure 4A, we have plotted the enthalpies versus the concentration for the different processes resolved. The enthalpies associated with the dilution step are rather small and will not be considered any further. Instead, we focus mainly on the slow process and $\Delta H_{\mathrm{dis}}$. At a high dilution, we find $\Delta H_{\mathrm{dis}}=-1.4$ $kJ{\mathrm{mol}}^{-1}$. We note that aggregate dissolution involves the transfer of peptide molecules from the aggregates to a hydrated state in the bulk solvent. The corresponding enthalpy change in the hydrophobic effect generally has a significant temperature dependence, with an increase in ΔH with increasing *T*. Moreover, it typically changes sign around room temperature [27,28]. Thus, the magnitude of this contribution is possibly small in the present case, but can be of either sign. As will be discussed in more detail below, the dissolution also involves a protonation step. The peptide monomers below $c_{s}$ have an average net charge of +1, while in the aggregates, roughly 40% of the molecules have dissociated a proton to become neutral. The deprotonation step is expected to give a negative increment to $\Delta H_{\mathrm{dis}}$ [29]. A more detailed interpretation of $\Delta H_{\mathrm{dis}}$ is outside the scope of the present paper.

In Figure 4B, we present $k^{-1}$ for the slow dissolution process. At the lowest concentration, we find $k^{-1}=30$ s, which we identify with the infinite dilution value, $k_{0}^{-1}$. $k^{-1}$ then increases as the concentration is increased. For a reversible process, where the net dissolution rate is the difference between detachment and attachment rates, we expect $k^{-1}=k_{0}^{-1}{\left(c_{s}-c\right)}^{-1}$ [30]. This functional form describes the lower concentrations with $c_{s}\approx 5$ mM reasonably well, as shown by the broken line in Figure 4B. However, it cannot describe the whole concentration range. In fact, the slow dissolution process is observed up to c≈8 mM.

The slow mode, associated with dissolution, is observed approximately up to 8 mM, which is higher than the $c_{s}\approx 5$ mM estimated from light scattering. However, when it comes to the slow mode, the higher concentrations are less reliable, as $k^{-1}$ approaches 1 h, which is the waiting time between injections, and consequently the dissolution process is not fully complete before the next injection. Furthermore, we note that α varies from 10 at low concentrations diverging towards 1 at high concentrations, however, we do not further interpret the meaning of this, as the main part of the lag phase is hidden in the $\Delta P_{\mathrm{dil}}$ and therefore hard to determine.

A striking observation is that the peptide dissolution is very slow. If the dissolution process was diffusion limited, we would expect the particles to be dissolved within milliseconds to seconds at lower peptide concentrations [30]. We may, for example, compare this with the diffusion-limited dissolution of a spherical aggregate at infinite dilution for which a simple analytical expression exists: $R\left(t\right)={\left(R_{0}^{2}-2Dvc_{s}t\right)}^{1/2}$ [30,31]. Here, *R* is the radius at time *t*, $R_{0}$ is the initial radius at t=0, *D* is the molecular self–diffusion coefficient in the solvent, v=550 cm${}^{3}$ is the monomer molar volume, and $c_{s}$ is, again, the monomer solubility. In this case, the radius becomes zero at $t=R_{0}^{2}/2Dvc_{s}$. The monomer diffusion coefficient of A${}_{6}$K has been measured to $3\times 10^{-10}$ m${}^{2}$s${}^{-1}$ at room temperature [32]. Assuming a similar, but slightly smaller value, $D=2\times 10^{-10}$ m${}^{2}$s${}^{-1}$, for the slightly larger A${}_{8}$K, and furthermore $R_{0}=100$ nm, and $c_{s}=2$ mM, we obtain that the radius should have become zero at t=9 ms. This should be compared to t≈2 min, observed here at high (infinite) dilution ($k^{-1}=30$ s). Clearly, the dissolution process in the present system is far from diffusion-limited, but rather reaction-limited, involving a free-energy barrier.

The dissolution of solid particles is typically a diffusion controlled process [30,33]. Therefore, what could be a possible barrier to dissolution here? The molecular pathway of dissolution can be complex and involve more than one process. In the related A${}_{6}$K system, the peptides also self-assemble into laminated β-sheets, although not into ribbons but into a cylindrically bent monolayer, forming hollow tubes. The dissolution process of the tubes appeared to initially involve a separation of the laminated β–sheets, followed by a dissolution of the β-sheets themselves [14]. The dissolution kinetics were not studied in detail in that paper, but it was noted that β-sheets were still observed after 5 min.

As the origin of the slow dissolution kinetics, we propose the necessary simultaneous breaking of more than one hydrogen bond from a β-sheet, before they can be replaced by peptide water hydrogen bonds. In fact, one way in which these peptide aggregates differ from most other solid particles is by their high density of hydrogen bonds. In an A${}_{8}$K β–sheet, there is the possibility of nine hydrogen bonds per molecule. Each hydrogen bond has a strength of roughly 5–10 $k_{B}$T [34,35]. The molecular dissolution of peptide molecules from a β–sheet involves replacing β-sheet hydrogen bonds with peptide–water hydrogen bonds. These are of similar strength, and the replacement is therefore not expected to give a significant contribution to $\Delta H_{\mathrm{dil}}$ [36,37]. However, it may strongly affect the kinetics. It is in fact reasonable that more than one hydrogen bond needs to be broken, due to steric hindrance, in order to make the proper replacements with water molecules. In this case, the barrier for detachment can indeed have a magnitude on the order of 10 $k_{B}$T or more.

**Figure 5:**
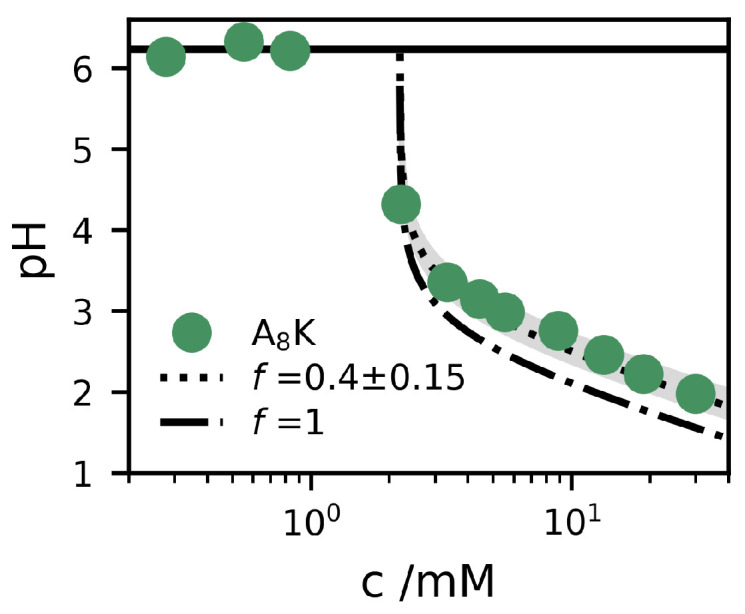
Measured pH (filled circles) as a function of the concentration, *c*, of A${}_{8}$K. Two model calculations of the pH are alsow shown, based on Equation (6), using $c_{s}=2.0$ mM and f=0.4 (dotted) and f=1 (dashed). The shaded grey area corresponds to the interval f=0.4±0.15. The pH=6.3 of the pure water solvent is shown as a solid line.

The solution pH was measured for different concentrations below and above $c_{s}$, and the results are presented in Figure 5. The experimental data show some particular features. First of all, for $c<c_{s}$, the pH ≈6.3 is essentially the same as that of pure water (with some dissolved CO${}_{2}$). This means that the A${}_{8}$K is a neutral salt and not a net acid or base. This implies that we can write the compound as PH${}^{+}$TFA${}^{-}$, where P stands for the A${}_{8}$K peptide. The dry compound is a TFA salt, but with only one TFA counterion, because the carboxyl group is neutralized by one of the two ammonium groups. Secondly, the pH drops above $c_{s}$, which means that the peptides deprotonate when they self-assemble. If every peptide drops a proton, the aggregates would be electroneutral and the process can be seen as producing TFA acid, H${}^{+}$TFA${}^{-}$, which can be considered a strong acid (pK${}_{a}\approx 1$ [38]). We would then have [H${}^{+}$]$=c-c_{\mathrm{mon}}$, where $c_{\mathrm{mon}}$ is the monomer concentration, and $c-c_{\mathrm{mon}}$ is hence the aggregate concentration. At equilibrium, we expect $c_{\mathrm{mon}}=c_{s}$. In Figure 5, we show the expected pH profile for this scenario, pH$=-\log\left(c-c_{s}\right)$. As can be seen, this curve slightly underestimates the pH, indicating that only a fraction *f* of the peptides are deprotonated when they self–assemble. In this case, we have

(6) $$\mathrm{pH}=-\log\left(\left(c-c_{s}\right)f\right)$$

Figure 5 also shows two model calculations of the pH for $c>c_{s}=2.0$ mM, according to Equation (6), for f=0.4 and 1, respectively. As can be seen, the data are well described by the curve with a constant f=0.4.

If we consider the peptide self-assembly as a precipitation of a new phase, then the deprotonation is not at all surprising. When two phases coexist, a titratable compound does not necessarily have the same protonation state in the two phases. Prime examples of this are the so called “acid–soaps” [39].

We obtain somewhat different values of the monomer solubility, in this study, depending on the experiment. The pH measurements are consistent with $c_{s}=2$ mM, while the ITC data indicate that $c_{s}$ rather is 8 mM. On the other hand, light scattering data suggest $c_{s}=5$ mM. The reason for this discrepancy is presently not clear.

## 3. Materials and Methods

### 3.1. Materials

Trifluoroacetate (TFA) salts of A${}_{8}$K peptides were purchased from CPC Scientific, Inc., with a purity of 97.4%. They were used without further purification. Samples were prepared by dissolving peptides in heavy water, D${}_{2}$O (99.8% isotope purity), obtained from Armar Chemicals. Heavy water was used for consistency with a related study involving NMR experiments [40]. Prior to sample preparation, the D${}_{2}$O was filtered using a GHP Acrodisc 25-mm syringe filter with a 0.2 μm GHP membrane. For conversion from the weight-volume fraction, *w*/*v*% to molar concentrations, the values used were 1.107 $g\;mL^{-1}$ for D${}_{2}$O density, and 829 $g\;\mathrm{mo}l^{-1}$ for the molecular weight of A${}_{8}$K with one TFA counterion. The mass density of A${}_{8}$K is 1.5 $g\;cm^{-3}$ [19].

### 3.2. Isothermal Titration Calorimetry

ITC measurements were performed on a Malvern MicroCal VP-ITC system using a reference power of 15 $\mathrm{cal}\;s^{-1}$ and a constant stirring speed of 307 rpm. At the start of every measurement, a null injection of 2 μL was performed over 4 s. After that, 27 consecutive 10 μL injections of an A${}_{8}$K stock solution (30 mM) were made into either pure solvent or a 4.4 mM A${}_{8}$K solution. The injection time and injection spacing were set to 30 s and 1 h, respectively. Water in water measurements were run between every sample to ensure a clean sample cell. These contributions were found to be negligible in comparison to the sample signal. All ITC measurements were performed at 25 ${}^{{}^{\circ}}C$.

Before further analysis of the acquired ΔP signal, the raw signal was corrected for slight baseline drifts using in-house code. The peaks in the ΔP signal were corrected by a first-degree polynomial between the data points, determined as the peak start and the peak finish. These boundaries were defined as when |ΔP/dt|, for the first and the last time, exceeded a threshold value of $2\times 10^{-3}$ $\mu J\;s^{-1}$. The rest of the signal was corrected by a third-degree polynomial. To further determine ΔH, the corrected ΔP trace of the various signal contributions was integrated.

## 4. Conclusions

To summarize, we have shown that ITC experiments can be useful to investigate not only the thermodynamics, but also the dissolution kinetics of peptide aggregates in solution. It is concluded that the aggregate dissolution, when diluting below the monomer solubility, $c_{s}$, is strikingly slow. Reaction-limited dissolution is very rare [33]. We propose here that the slow reaction-limited dissolution is most likely related to the necessary breaking of several β-sheet hydrogen bonds before they can be replaced by hydrogen bonding to water in the process of molecular detachment from β-sheet aggregates. This is, in fact, expected to be a general property of high-density hydrogen-bonded compounds. In addition, we found that the peptides partly deprotonate upon aggregation. pH measurements indicate that approximately 40% of the peptides deprotonate and become neutral. We argue that this is a general behavior when precipitating titratable compounds and draw the similarity to acid soaps. The present finding should be of significant importance for various applications of peptide self-assembly where, e.g., controlled dissolution and protonation state is important, such as in peptide drug delivery [41].
